# Associations Between Mineral Composition and Aflatoxin B1 in Maize (*Zea mays* L.) Seeds: Toward Contamination Indicators and Food Safety

**DOI:** 10.3390/foods14203552

**Published:** 2025-10-18

**Authors:** Dragana Bartolić, Rada Baošić, Jelena Mutić, Mira Stanković, Dragosav Mutavdžić, Nevena Preradović, Saša Krstović, Ksenija Radotić

**Affiliations:** 1 Institute for Multidisciplinary Research (IMSI), University of Belgrade, 11030 Belgrade, Serbia; mira.mutavdzic@imsi.bg.ac.rs (M.S.); gane@imsi.bg.ac.rs (D.M.); nevena@imsi.bg.ac.rs (N.P.); xenia@imsi.bg.ac.rs (K.R.); 2 Center for Green Technologies, Institute for Multidisciplinary Research (IMSI), University of Belgrade, 11030 Belgrade, Serbia; 3 Faculty of Chemistry, University of Belgrade, Studentski trg 12-16, P.O. Box 51, 11158 Belgrade, Serbia; rbaosic@chem.bg.ac.rs (R.B.); jmutic@chem.bg.ac.rs (J.M.); 4 Department of Animal Science, Faculty of Agriculture, University of Novi Sad, 21102 Novi Sad, Serbia; sasa.krstovic@stocarstvo.edu.rs

**Keywords:** AFB1, maize seed, ICP-OES, ICP-QMS, PCA, correlation analysis, food safety

## Abstract

Maize (*Zea mays* L.), a globally important cereal crop, is often threatened by aflatoxin contamination, compromising seed quality, nutritional value, and food safety. This study investigated the distribution of macro- and microelements in inner and outer seed fractions of maize with varying aflatoxin B1 (AFB1) levels to identify potential elemental markers of contamination. Macro- and microelements were quantified using ICP-OES and ICP-QMS, and principal component and correlation analyses were applied to explore interelement relationships and sample separation. The inner fraction was enriched in metabolically active elements such as K, Mg, Fe, Zn, Mn, and Ni, while the outer fraction contained higher Na, Ca, Cr, and Co, supporting structural integrity and defense. Strong positive correlations were observed between Mn and Zn in the inner fraction (r = 0.818), as well as between Cr and Zn (r = 0.82) and Co and Zn (r = 0.797) in the outer fraction, whereas Na and Zn showed a negative correlation in the inner fraction (r = −0.739). Na exhibited a nonlinear relationship with AFB1, suggesting complex regulatory mechanisms. Increasing AFB1 concentrations affected elemental composition, with dynamic changes in Cr, Mn, Zn, and Co and reductions in K, reflecting adaptive responses at low toxin levels and disrupted metal homeostasis at higher contamination. Strong associations of Mn, Zn, and Cr with AFB1 indicate their potential as contamination markers. These findings highlight compartment-specific mineral dynamics and their relevance for seed resilience and food safety.

## 1. Introduction

Maize (*Zea mays* L.) is one of the most important staple crops for both human and animal consumption worldwide, making the quality of maize grains critically important. Fungal pathogens cause crop losses and pose food safety issues. The fungal pathogen *Aspergillus flavus* (*A. flavus*) infects cereal crops, resulting in the contamination of seeds with aflatoxins (AFs), toxic secondary metabolites. According to reports by the Food and Agriculture Organization (FAO), approximately 25% of the global maize crop is contaminated annually, resulting in economic losses estimated in the billions of dollars [1]. Mycotoxin contamination in seeds can be influenced by a wide range of environmental and agronomic factors, including temperature, humidity, and storage conditions, among others [2]. These toxins pose serious health risks to both humans and livestock and significantly reduce the value of grains as animal feed and export food commodities [3,4]. Given these risks, the existence and implementation of effective veterinary sanitary and food safety regulations at both national and European levels are essential for protecting public health and ensuring food quality [5,6,7].

Seed quality can be negatively affected by mycotoxins, leading to reduced germination and impaired subsequent growth and development of the plant. These toxins may lead to oxidative damage in plants [8]. The defense mechanisms of plants are crucial for coping with biotic stress induced by fungal pathogens and their toxins. This innate defense capacity is closely linked to the plant’s nutritional status, which plays a key role in modulating disease resistance mechanisms. As highlighted by Tripathi et al. [9], plant mineral nutrition is intricately associated with disease resistance, forming an essential component of sustainable crop protection strategies. The macroelements K, Ca, and Mg, along with the micronutrients Mn, Fe, Zn, and Cu, play a significant role in promoting healthy plant growth and imparting disease resistance. Balanced mineral nutrition plays a key role in plant defense by activating redox enzymes and enhancing resistance through improved root exudation and shaping the composition of the microbial community. They also play a crucial role in the biosynthesis of lignin and phytoalexins, as well as in regulating various metabolic processes essential for plant stress responses [9]. Lignification of the seed coat acts as a primary defense mechanism against pathogen invasion and toxin progression [10,11,12], typically triggered by redox changes following ROS accumulation [13]. Bartolić et al. showed that AFB1-contaminated *Zea mays* L. seeds have enhanced content of lignin and organic free radicals [14]. Atanasova-Penichon et al. [15] demonstrated that, depending on the type of phenolic compound, the production of certain mycotoxins can be either stimulated or inhibited. Accumulation of specific polyamine conjugates and changes in total phenolic content were observed in AFB1-stressed *Zea mays* L. seeds [16]. Phenolic compounds can act as chelators of transition metal ions (e.g., Cu^2+^, Fe^2+^, Zn^2+^, Mn^2+^), thereby influencing the redox balance within plant cells and regulating the expression of antioxidant enzymes. On the other hand, depending on their interaction with redox-active metals, they can act as antioxidants or prooxidants by ROS levels [17,18]. Lipid metabolism may also be closely associated with both AFB1 synthesis and the elemental composition of seeds. Lipid desaturation promotes AFB1 synthesis in *A. flavus* [19]. Some elements interact with lipid desaturation in maize seeds and thereby may influence AFB1 biosynthesis [20].

Metals, already present or taken up during plant growth, affect the level of mycotoxin contamination in seeds, through promoting or inhibiting the growth of fungi that produce mycotoxins. Pro-oxidant metals (e.g., Fe, Cu) can cause oxidative stress, which may reduce mycotoxin production by fungi [21]. Some metals (e.g., Zn, Mn) are essential for fungal metabolism and may increase fungal growth and aflatoxin biosynthesis if available in moderate amounts [21,22,23]. Essential nutrients such as nitrogen, potassium, manganese, and zinc have specific effects on plant-pathogen interactions, with some strengthening resistance and others potentially increasing susceptibility and mycotoxin production. Furthermore, elements are not distributed uniformly throughout the grains: some, such as Fe and Zn, are concentrated in the embryo, while others, like Ca, accumulate substantially in the maternal tissues of the pericarp [24,25,26,27,28].

Thus, metal composition of seeds can affect the fungal communities and influence susceptibility to contamination. On the other side, fungal infection can alter plant physiology, potentially affecting nutrient (and metal) uptake.

Unlike prior studies, this research investigates natural maize samples, determining the actual metal content and contamination of the seeds’ inner and outer fraction, which offers more realistic and applicable data for food safety and agricultural monitoring. Simultaneous consideration of micro- and macroelements in seeds and the integrated use of multivariate analysis (using principal component analysis and correlation analysis) further distinguishes this study, offering a more comprehensive understanding of the complex interactions that influence mycotoxin contamination. Understanding this relationship could contribute to better risk assessment and management strategies for aflatoxin contamination in maize production, ultimately improving food safety and crop quality.

## 2. Materials and Methods

### 2.1. Reagents and Chemicals

Nitric acid (65%, *v*/*v*) and hydrogen peroxide (30%, *v*/*v*) were of analytical grade and supplied by Merck (Darmstadt, Germany). Millipore Simplicity 185 System (incorporating dual UV filters, 185 and 254 nm) was used to prepare ultra-pure water. Stock solution containing 0.5000 g L^−1^ of major elements (VHG Labs, Manchester, NH, USA) and semi-quantitative stock solution containing 0.0100 g L^−1^ of minor elements (Alfa Aesar, Ward Hill, SAD) were used to prepare intermediate multi-element standard solutions for determination on ICP-OES and ICP-QMS, respectively. Certified reference material ERM—CD281 (rye grass) IRMM (Institute for Reference Materials and Measurements) was used to verify the accuracy and precision of the ICP-OES and ICP-MS instruments [29]. The results of the analysis showed good agreement with the certified levels (Supplementary Table S1). Analytical performance of each element determination is listed in Supplementary Table S2.

### 2.2. Sample Preparation

The maize (*Zea mays* L.) seeds with known AFB_1_ concentrations were kindly provided by the Faculty of Agriculture, University of Novi Sad, Serbia. The samples were collected under field conditions and cover the relevant exposure range found in contaminated maize. The AFB_1_ concentrations were determined using the modified Association of Official Analytical Chemists method 980.20 [30]. Aflatoxin B_1_ determination involved using immunoaffinity column cleanup and high-performance liquid chromatography (HPLC) with fluorescence detection. Ground samples were extracted with methanol-water (70:30, *v*/*v*) and filtered. Filtered and diluted extracts were then run through the immunoaffinity columns (Vicam, Milford, MA, USA) and washed with ultrapure water before methanol elution. The eluate was evaporated under a gentle stream of nitrogen. AFB_1_ was derivatized with trifluoroacetic acid and n-hexane at 35 °C before being reconstituted in methanol/water/acetonitrile (60:20:20, *v*/*v*/*v*). A sample volume of 20 µL was injected into Agilent 1260 Infinity HPLC system (Agilent Technologies, Santa Clara, CA, USA) using a mixture of methanol/water/acetonitrile (60:20:20, *v*/*v*/*v*) as a mobile phase on a 4.6 × 150 mm, 5 μm C18 column (Agilent Technologies, Santa Clara, CA, USA) at a flow rate of 1 mL·min^−1^ and detection on fluorescence detector (excitation λ = 360 nm and emission λ = 440 nm).

To conduct the analysis, naturally contaminated seeds with AFB_1_ concentrations of 6.75, 13.26, 17, 51.51, 61, 105, 151.94, 177.42, 248, 299, and 308.13 μg kg^−1^, as well as uncontaminated seeds (control), were selected. The samples were ground using a laboratory mortar grinder (Fritsch Pulverisette 2, Berlin, Germany). The inner fraction (IF—mainly starchy endosperm) and the outer fraction (OF—mainly pericarp and aleurone) were separated using appropriate laboratory sieves (250 µm mesh).

Microwave digestion of the samples (~200 mg) was performed using 5 mL of HNO_3_ (65%, *v*/*v*) and 2 mL of H_2_O_2_ (30%, *v*/*v*) in a microwave oven (Berghof Speedwave, Berghof, Germany), applying a temperature program with ramping to 170 °C (10 min, hold 10 min, pressure 30 bar) and then to 200 °C (10 min, hold 20 min, pressure 35 bar) at 100% power. After a cooling period, the samples were quantitatively transferred into a volumetric flask of 25 mL and diluted with ultra-pure water. For analyses at ICP-QMS, the corn samples were further diluted 5 times.

### 2.3. ICP-OES and ICP-QMS

Macroelements and Fe were measured by ICP-OES (model 6500 Duo, Thermo Scientific, Abingdon, UK). ICP-OES analysis was performed under standard operating conditions (RF power 1150 W, Principal argon flow rate 12 L/min, auxiliary argon flow rate 0.5 L/min, nebulizer argon flow rate 0.5 L/min, sample flow rate 1 mL/min), using a concentric nebulizer and cyclonic spray chamber, and equipped with a CID86 detector. The following wavelengths (nm) were selected for macroelements: Ca (317.9 nm), K (766.4 nm), Na (589.5 nm), Mg (280.2 nm), and Fe (238.2 nm). The entire system of ICP-OES was controlled using Iteva software.

ICP-QMS analysis was performed under standard operating conditions (RF power 1548 W, gas flows 13.9, 1.09 and 0.8 L/min; acquisition time 3 × 50 s; points per peak 3; dwell time 10 ns; pulse detector mode). The following isotopes were selected for microelements: ^55^Mn, ^59^Co, ^62^Ni, ^63^Cu, ^66^Zn. Concentrations of microelements were determined with ICP-QMS (iCAP Q, Thermo Scientific X series 2). The entire system of ICP-QMS is controlled using Qtegra Instrument Control Software.

### 2.4. Data Analysis

Comparison of mean microelement and macroelement contents among seed fractions with different AFB1 concentrations was performed using a two-way ANOVA (balanced design, n = 3), followed by Duncan’s post hoc test, with a significance level set at 5%.

Principal Component Analysis (PCA) was employed as a multivariate statistical technique to reduce the dimensionality of the dataset to investigate how variations in aflatoxin contamination levels influence the elemental profiles and gain insight into complex relationships among the elemental profiles. This goal was achieved by transforming the original correlated variables into a smaller set of uncorrelated principal components, two in this case, and constructing a biplot. Before applying Principal Component Analysis (PCA), all variables representing elemental profiles were standardized to eliminate the influence of differences in measurement scales and ensure comparability across parameters. PCA was then conducted using XLSTAT 2018 software (Addinsoft Inc., New York, NY, USA). Linear and non linear regression was performed in OriginPro 2018 using the weighted least squares method, taking into account the standard errors of the measurements.

## 3. Results

### 3.1. Contents of Macro- and Microelements

The contents of macroelements (K, Ca, Mg, Na) and microelements (Fe, Zn, Cu, Mn, Co, Ni, Cr) in the inner and outer fractions of *Zea mays* L. seeds with varying aflatoxin B1 (AFB1) levels were determined using ICP-OES and ICP-QMS (Table 1). Results of comparisons of mean values of micro- and macroelements between different AFB1 concentrations separately in IFs and OFs are shown in Supplementary Table S3. The inner seed fraction contained higher concentrations of K, Mg, Fe, Zn, Mn, and Ni than the outer fraction.

### 3.2. Principal Component Analysis and Correlation Analysis

**Figure 1:**
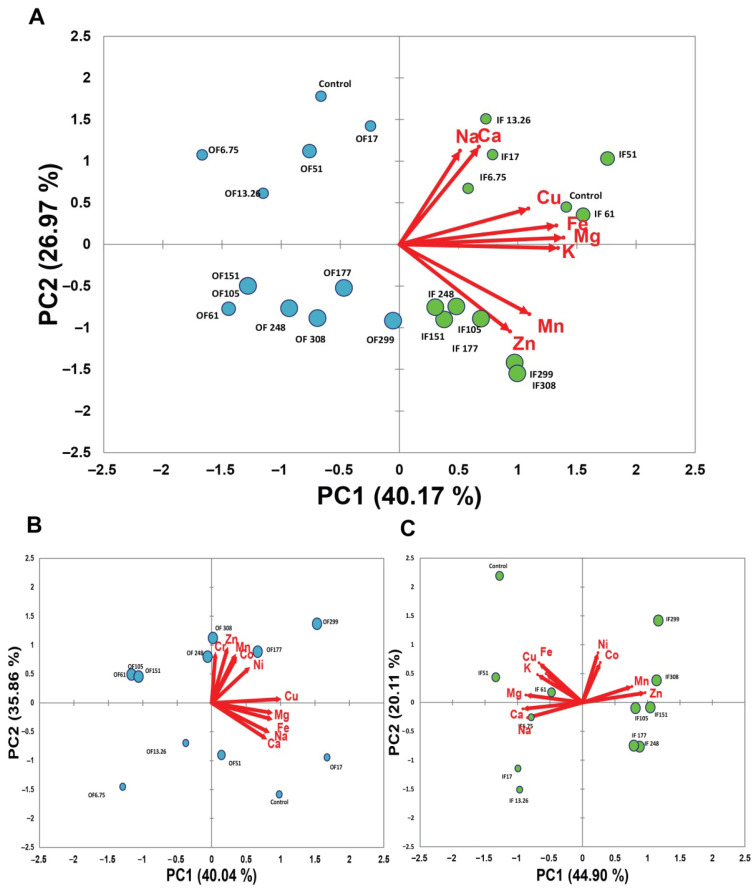
Biplots (PC1 vs. PC2) of individual elemental content across seed fractions with varying levels of AFB1 contamination (**A**), and within each fraction separately—outer (**B**) and inner (**C**). Outer and inner seed fractions are marked in blue and green, respectively. Circle size corresponds to AFB1 concentration.

To explore underlying patterns and assess the association of elemental profiles with AFB1 contamination, PCA was subsequently applied to macro- and microelement content of seeds with varying contamination levels to evaluate their separation based on elemental composition. The biplot in Figure 1A shows a clear separation of the two fractions, outer and inner. By analyzing the directions of the vectors representing all elements except for Na and Ca, it is observed that the inner layer has higher “concentrations” of these elements than the outer layer. Vectors Na and Ca show the least presence of these elements in the outer layer at higher aflatoxin concentrations. The biplot in Figure 1B shows that an increase in the level of aflatoxin leads to an increase in the level of Cr, Zn, Mn, Ci, and Ni, while this linear trend cannot be observed for the other elements. The biplot in Figure 1C indicates that an increase in aflatoxin levels leads to an increase in Mn and Zn levels and a decrease in Ca, Mg, and Na levels.

Correlation coefficient values higher than 0.5 were used for the interpretation of the correlation analysis. Na showed a strong positive correlation with Ca (around r = 0.94) and Mg (0.607) in both seed fractions. It can be observed that Na is negatively correlated with Zn (−0.739) in the inner fraction, while showing positive correlations with Cu (0.790) and Fe (0.836) in the outer fraction seeds. As can be seen from Table 2, the correlation analysis for Ca shows a similar pattern to that of Na. A significant positive correlation was observed between K and Fe concentrations (r = 0.630), but only in the inner fraction of the seed. Magnesium exhibited significant correlations with a larger number of elements across both the inner and outer seed fractions. In the inner seed fraction, Mg showed strong positive correlations with Cu (r = 0.641), Fe (r = 0.644), Ca (r = 0.663), and Na (r = 0.607), while exhibiting a strong negative correlation with Zn (r = −0.715).

### 3.3. Relationship Between Macro- and Microelements and AFB1 Concentration

**Figure 2:**
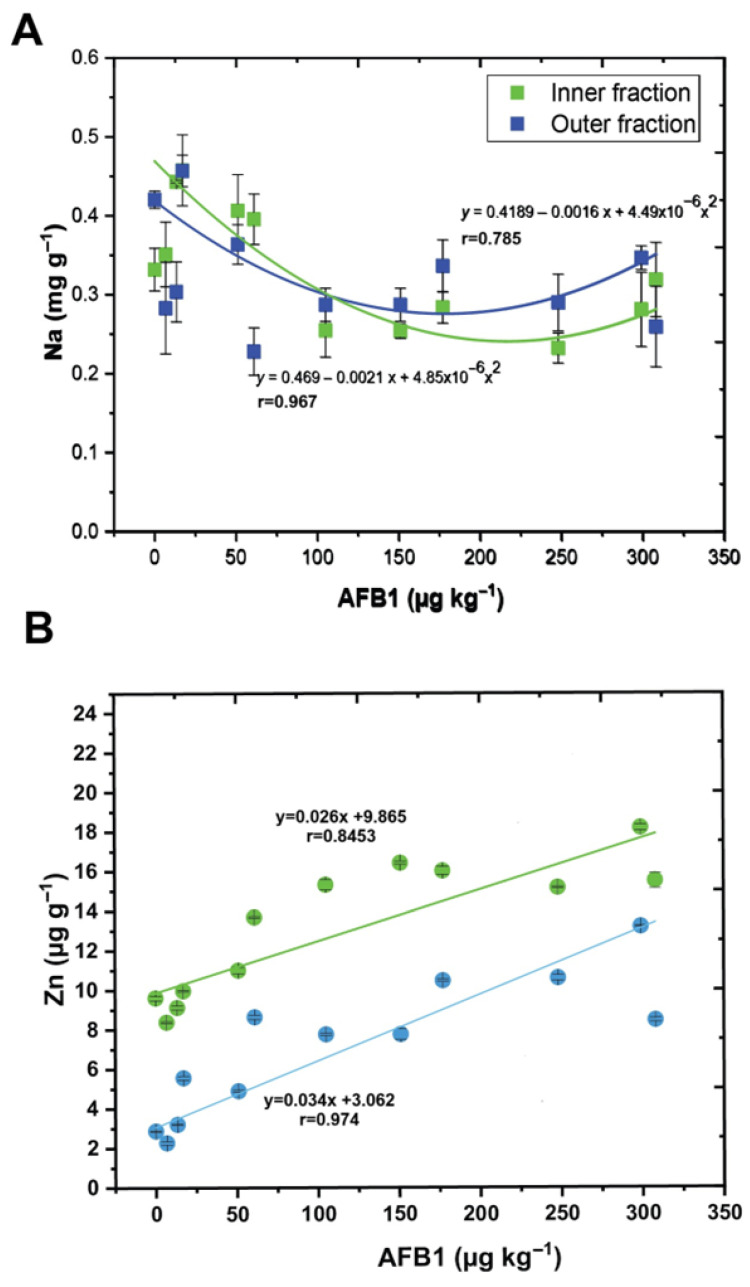
An example of two selected elements (macro- and microelements) and AFB1 concentrations in the inner and outer seed fractions. (**A**) Nonlinear second polynomial relationship between Na and AFB1 concentrations, and (**B**) linear relationship between Zn and AFB1 concentrations. r—correlation coefficient. (±SD of three measurements).

Further investigation of the relationship between macro- and microelement concentrations and AFB1 levels was elaborated. The results show a significantly strong linear relationship between Zn content and AFB1 concentration in the inner seed fraction (r = 0.8453), as well as in the outer fraction (r = 0.974) (Figure 2B). A significantly strong positive linear relationship was established between Mn content and AFB1 concentration in both seed fractions (r = 0.8). As shown in Figure 2A, a significant nonlinear (second-degree polynomial) relationship was observed between AFB1 concentration and Na content in both fractions. The relationship was stronger in the inner fraction (r = 0.967) than in the outer fraction, which exhibited a moderate fit (r = 0.785). On the other hand, AFB_1_ concentration has a significant effect on Ca content in the inner seed fraction, following a nonlinear trend (r = 0.83), whereas no such relationship was observed in the outer fractions (r = 0.38). The results show a statistically significant negative linear relationship between K content and AFB1 concentration (r = −0.709) in the inner seed fraction (while no statistically significant relationship was observed in the outer seed fraction. No clear relationship was observed between variations in Mg, Co, and Ni concentrations and aflatoxin content. The relationship between AFB1 concentration and Cr levels showed a positive relation to 61 µg kg^−1^ AFB1. Algül and Kara showed a strong positive relation between Cr and total Aflatoxin and Aflatoxin B1, in the concentration range from 0.17 to 12.87 µg kg ^−1^ [31].

## 4. Discussion

The concentrations of macro- and microelements observed in this study are generally consistent with those reported in the literature for maize seeds. The mineral composition of both seed fractions (Table 1) was characterized by a predominance of potassium (K) among the macronutrients [32]. Prasanthi et al. [33] reported similar Mg and Ca concentrations, with Ca consistently at lower levels compared to other macronutrients, which agrees with our findings. Sodium (Na) concentrations in both inner and outer fractions were also in line with values typical for maize and other cereals, where Na generally occurs at relatively low levels [32,33]. Variations in macronutrient content in seeds can depend on factors such as genetic background, environmental conditions, agricultural management, and abiotic or biotic stresses affecting the plant [5]. The higher concentrations of K, Mg, Fe, Zn, Mn, and Ni in the inner fraction (Table 1) suggest a greater metabolic activity in this tissue compared to the outer fraction [34].

A clear separation between the inner and outer seed fractions can be observed for all analyzed samples along the PC1 axis (Figure 1A). Since this component is positively correlated with the contents of Fe, Cu, K, and Mg, it indicates that the inner fractions contain higher levels of these elements compared to the outer fractions. The non-uniform distribution of elements indicates their specific role in plant metabolism and defense. Metabolically active tissues (embryo, endosperm, aleurone) accumulate higher amounts of such elements, whereas protective tissues (seed coat, pericarp) serve mainly as barriers [35].

The biplots in Figure 1 provide an integrated overview of the relationships between element concentrations and AFB1 content in the inner and outer seed fractions. Along the PC2 axis in Figure 1A, separation was observed between seed samples with lower and higher concentrations of AFB1. Zn and Mn are highly positively correlated in both seed fractions (Figure 1B,C, Table 2), indicating that Zn content varies similarly to Mn in relation to contamination levels. A strong positive linear relationship between Zn content and AFB_1_ concentration was observed in both seed fractions (Figure 2B), consistent with reports that Zn^2+^ ions stimulate aflatoxin biosynthesis [22,36]. The parallel variation of Mn with AFB1 levels likely reflects its role in activating phenylpropanoid enzymes, which are upregulated under biotic stress such as fungal infection [37,38]. Mn is involved in the synthesis of phenolic compounds and plant defense [38], and its increase, along with Zn, correlates strongly with AFB1 content (r = 0.818 in the inner fraction), suggesting their roles in physiological responses to fungal infection and mycotoxin-induced stress. Thus, Mn and Zn may serve as indicators of aflatoxin contamination in seeds. As shown in Figure 1, the samples with higher aflatoxin levels tended to have lower amounts of Na and Ca. This trend may indicate a protective role of Na and Ca, potentially through the stabilization of cell walls, maintenance of membrane integrity, and modulation of ion homeostasis under stress conditions [39,40,41,42,43,44,45,46]. Similar inverse relationships between Ca content and mycotoxin contamination have been reported in cereals, where Ca is implicated in strengthening structural barriers and signaling pathways that reduce pathogen invasion [42,47]. The strong correlation between sodium (Na) and calcium (Ca) observed in both seed fractions may reflect their complementary roles in maintaining ionic balance and stress response, particularly under the oxidative and physiological stress associated with aflatoxin contamination. Critical secondary messengers in plant defense and cell wall stabilization may also contribute to osmotic regulation and compensate for ionic imbalances [42,48]. The observed nonlinear response of Na to increasing levels of AFB1 (Figure 2A) can be explained by its critical role in maintaining cellular ion homeostasis and membrane stability, which may be disrupted by aflatoxin-induced oxidative stress. One possible explanation for this observation is that aflatoxin impairs membrane integrity and permeability, disrupting ion transport systems and inhibiting ATP-dependent ion pumps such as Na^+^/K^+^-ATPase [49,50]. On the other hand, a similar variation in Ca with AFB1 concentrations may suggest that its dynamics may be actively involved in the plant defense signaling pathways response to fungal invasion and toxin stress [51]. It is well known that Ca is a secondary messenger in signaling pathways triggered by abiotic and biotic stressors and contributes to cell wall reinforcement, regulation of programmed cell death in infected tissues to limit pathogen spread, and activation of defense-related genes. Huber and Graham showed that pathogens can affect membrane permeability and nutrient mobilization toward infected sites, which may lead to nutrient deficiencies or toxicities. Specifically, *Fusarium oxysporum* f. vasinfectum was reported to decrease the concentration K, Ca, and Mg in leaves. Together, suitable levels of Ca and Na may enhance plant resistance to fungal infections and reduce mycotoxin accumulation [52,53].

Correlation analysis (Table 2) suggests that Na may facilitate the accumulation or mobility of divalent cations such as Ca, Mg, Cu, and Fe, particularly in the outer seed fraction, with a strong positive correlation with Ca (r ≈ 0.94), indicating possible synergistic interactions or shared transport mechanisms. Conversely, the negative correlation between Na and Zn in the inner fraction (r = −0.739) points to a potential antagonism affecting Zn bioavailability, which could influence antioxidant defense and pathogen resistance. In contrast, K consistently showed higher concentrations in the inner fraction but lacked a significant correlation with AFB1, suggesting that its distribution is determined mainly by physiological roles—osmotic regulation, enzyme activation, and carbohydrate transport—rather than by mycotoxin accumulation [35,54,55].

In the outer seed fraction, the positive correlation between Fe and Na (Figure 1B) likely reflects their shared roles in structural and protective functions, accumulating in the pericarp to support barrier properties, oxidative stress resistance, and ion balance. Sodium may also influence Fe mobility or retention via shared transport mechanisms or by stabilizing local ionic conditions. The pronounced correlations between Cr and Zn and between Co and Zn in the outer fraction suggest potential co-accumulation or shared transport and binding mechanisms in the seed coat, possibly involving metal-binding proteins or cell wall constituents [56,57]. Cr, Co, and Zn were found at higher concentration in the samples with higher AFB1 content (Figure 1B). Such relationships for Cr and Co were absent in the inner fraction, supporting the hypothesis of compartment-specific metal interactions [58]. Cr content initially increased with low AFB1 levels, reflecting an adaptive hormetic response involving metal-binding proteins and oxidative stress defenses, plateaued possibly due to disrupted Cr-handling pathways [59,60], and rose again at higher AFB1 concentrations, consistent with reported correlations with total AFB1 in maize [30].

Interestingly, nickel (Ni) correlated with Zn and Cu only in the outer fraction and showed no significant association with AFB1 concentration, indicating that its accumulation is influenced more by metal–metal interactions than by contamination levels [61]. The decrease in Cu content in the inner seed fraction with increasing AFB1 levels may be part of a broader adaptive response to oxidative stress, involving complex interactions between metal homeostasis and mycotoxin-induced damage [43,62].

A positive correlation between K and Fe in the inner seed fraction likely reflects their roles in metabolically active tissues, co-regulated ion transport, and stress responses. Both elements contribute to redox balance, suggesting that they share regulatory pathways affected by stress. The observed negative relationship between K and AFB1 indicates that aflatoxin may disrupt membrane integrity or nutrient uptake, reducing K accumulation and impairing enzyme activation, osmotic regulation, and nutrient transport. In contrast, Mg levels in the outer layer showed no clear association with AFB1, suggesting stability of Mg-related functions under moderate toxin exposure. Many essential elements act as enzyme cofactors (Fe, Cu, Zn) or activators (Mn, Zn, Cu, Fe, Ca) and can influence secondary metabolite biosynthesis in fungi [63]. Fe and Cu are transition metals involved in redox reactions and can generate reactive oxygen species under stress conditions.

From a food safety perspective, these findings are relevant because the outer layers of cereal grains are often retained in whole-grain products or used in animal feed [64], both of which can be routes for human and livestock exposure to aflatoxins [65]. The observed co-occurrence of certain microelements (e.g., Zn, Mn, Cr, Co) with high aflatoxin levels in the seed coat raises questions about whether these elements are merely markers of contamination or actively influence fungal proliferation and toxin biosynthesis [66,67]. Moreover, since the outer fraction exhibited more pronounced metal–metal correlations and stronger associations with AFB1, monitoring its elemental composition could serve as an additional indicator of contamination risk [42]. On the other side, Na and Ca contents are inversely associated with AFB1 levels and may contribute to contamination resistance, while K appears to play a more constitutive role in seed physiology without a direct link to aflatoxin accumulation. Understanding these dynamics is critical for developing strategies aimed at biofortification and improving seed nutritional quality, which ultimately contributes to enhanced crop performance and food security.

Future work could include a larger number of field-collected AFB1-contaminated seed samples to improve data consistency and reliability.

## 5. Conclusions

Our study demonstrates that elemental distribution in *Zea mays* L. seeds is strongly compartment-specific, with the inner fraction enriched in K, Mg, Fe, Zn, Mn, and Ni, reflecting higher metabolic activity, while the outer fraction contains greater amounts of Na, Ca, Cr, and Co, supporting structural integrity and defense. Correlations among elements, including synergistic (Na–Ca, Cr–Zn) and antagonistic (Na–Zn) interactions, suggest complex regulatory mechanisms affecting nutrient availability and stress responses. AFB1 exposure alters mineral composition, with low toxin levels triggering adaptive responses (e.g., Cr, Mn) and higher concentrations potentially disrupting metal homeostasis. The accumulation patterns of Mn, Zn, and Cr, and their association with AFB1, indicate their potential as markers of aflatoxin contamination. These findings not only provide insights into seed physiology and defense but also have implications for food safety and nutritional quality, highlighting the need to monitor mineral composition in maize to ensure consumer protection. Future research should include other relevant mycotoxins, as well as explore their possible associations with micro- and macroelement composition.

## Figures and Tables

**Table 1:** Content of micro- (μg g^−1^) and macroelements (mg g^−1^) in the inner and outer fraction of seeds with varying AFB_1_ levels of contamination (n = 3).

	AFB1 (μg kg^−1^)	Cr (μg g^−1^)	Mn (μg g^−1^)	Co (μg g^−1^)	Ni (μg g^−1^)	Cu (μg g^−1^)	Zn (μg g^−1^)	Fe (μg g^−1^)	Ca (mg g^−1^)	K (mg g^−1^)	Mg (mg g^−1^)	Na (mg g^−1^)
Inner fraction	Control	0.75 ±0.016	6.88 ±0.031	0.15 ±0.001	2.34 ±0.03	7.7 ±0.053	9.61 ±0.112	27.64 ±0.382	1.46 ±0.105	3.43 ±0.189	1.5 ±0.021	0.33 ±0.027
	6.75	0.79 ±0.004	6.33 ±0.053	0.14 ±0.001	1.51 ±0.02	5.18 ±0.072	8.38 ±0.045	18.61 ±0.35	1.67 ±0.136	3.08 ±0.047	1.4 ±0.002	0.35 ±0.041
	13.26	0.74 ±0.01	7.14 ±0.041	0.12 ±0	1.17 ±0.007	5.88 ±0.019	9.12 ±0.099	14.57 ±0.289	1.9 ±0.077	2.97 ±0.176	1.33 ±0.014	0.44 ±0.002
	17.07	0.88 ±0.008	6.54 ±0.092	0.13 ±0.001	1.06 ±0.012	5.96 ±0.099	9.97 ±0.043	14.27 ±0.075	1.73 ±0.058	2.95 ±0.161	1.45 ±0.008	0.46 ±0.045
	51.51	0.98 ±0.01	8.06 ±0.077	0.13 ±0.001	1.45 ±0.006	8.78 ±0.154	11 ±0.159	22.68 ±0.271	1.91 ±0.098	3.15 ±0.308	1.44 ±0.004	0.41 ±0.046
	61	1.01 ±0.028	9.76 ±0.115	0.13 ±0.001	1.57 ±0.025	7.08 ±0.034	13.69 ±0.043	22.03 ±0.203	1.79 ±0.069	2.76 ±0.261	1.41 ±0.005	0.4 ±0.032
	105	0.86 ±0.022	10.47 ±0.083	0.12 ±0.002	1.6 ±0.011	4.86 ±0.056	15.31 ±0.262	16.31 ±0.08	0.92 ±0.076	3.21 ±0.158	1.26 ±0.018	0.26 ±0.034
	151.94	0.87 ±0.023	8.12 ±0.037	0.13 ±0.001	1.93 ±0.043	5.45 ±0.037	16.42 ±0.081	12.13 ±0.032	0.98 ±0.081	2.97 ±0.201	1.17 ±0.026	0.25 ±0.009
	177.42	0.87 ±0.023	9.5 ±0.026	0.13 ±0.001	1.57 ±0.012	5.38 ±0.064	16.01 ±0.204	12.36 ±0.08	1.05 ±0.06	2.36 ±0.065	1.37 ±0.019	0.28 ±0.02
	248	0.87 ±0.003	7.93 ±0.213	0.13 ±0.001	1.42 ±0.011	5.5 ±0.025	15.16 ±0.046	12.06 ±0.222	1.02 ±0.171	2.63 ±0.094	1.32 ±0.018	0.23 ±0.019
	299	0.97 ±0.02	10.89 ±0.082	0.16 ±0	2.46 ±0.024	6.07 ±0.097	18.19 ±0.152	14.24 ±0.052	1.22 ±0.065	2.89 ±0.135	1.26 ±0.022	0.28 ±0.047
	308.13	1.3± 0.016	11.15 ±0.21	0.16 ±0	1.29 ±0.02	5.04 ±0.081	15.5 ±0.384	17.44 ±0.345	1.2 ±0.07	2.77 ±0.298	1.24 ±0.017	0.32 ±0.047
Outer fraction	Control	0.68 ±0.013	4.19 ±0.054	0.14 ±0.001	1.61 ±0.011	5.87 ±0.028	2.88 ±0.029	11.13 ±0.101	1.85 ±0.068	1.42 ±0.08	0.68 ±0.011	0.42 ±0.011
	6.75	0.66 ±0.005	3.25 ±0.031	0.12 ±0.001	0.71 ±0.029	3.91 ±0.014	2.27 ±0.088	7.28 ±0.158	1.18 ±0.199	1.14 ±0.224	0.54 ±0.002	0.28 ±0.058
	13.26	0.74 ±0.016	4.78 ±0.058	0.12 ±0.001	1.95 ±0.016	4.66 ±0.074	3.21 ±0.022	7.39 ±0.162	1.19 ±0.082	1.72 ±0.109	0.61 ±0.014	0.3 ±0.038
	17.07	0.83 ±0.013	4.9 ±0.081	0.15 ±0.001	1.7 ±0.028	6.45 ±0.09	5.56 ±0.098	10.73 ±0.128	2.03 ±0.048	1.37 ±0.217	0.82 ±0.006	0.46 ±0.02
	51.51	0.88 ±0.026	4.67 ±0.061	0.12 ±0.001	0.96 ±0.009	5.39 ±0.066	4.9 ±0.049	8.28 ±0.064	1.57 ±0.13	1.5 ±0.221	0.66 ±0.012	0.36 ±0.025
	61	0.88 ±0.006	4.75 ±0.103	0.15 ±0.001	1.48 ±0.018	4.17 ±0.018	8.64 ±0.108	7.67 ±0.193	0.78 ±0.093	1.18 ±0.242	0.46 ±0.007	0.23 ±0.03
	105	0.99 ±0.019	5.25 ±0.054	0.14 ±0.001	1.63 ±0.042	4.33 ±0.006	7.76 ±0.05	7 ±0.243	0.88 ±0.092	1.22 ±0.307	0.42 ±0.012	0.29 ±0.021
	151.94	1.01 ±0.018	7.54 ±0.042	0.15 ±0.001	1.87 ±0.016	6.08 ±0.059	13.83 ±0.275	9.65 ±0.322	0.92 ±0.026	2.24 ±0.115	0.73 ±0.008	0.3 ±0.021
	177	1± 0.03	6.47 ±0.072	0.16 ±0.001	2.6 ±0.035	6.08 ±0.012	10.47 ±0.061	8.21 ±0.144	1.31 ±0.024	1.26 ±0.19	0.69 ±0.007	0.34 ±0.033
	248	0.87 ±0.008	5.48 ±0.031	0.16 ±0.001	2.8 ±0.04	5.34 ±0.13	10.6 ±0.14	7.15 ±0.274	0.99 ±0.101	1.16 ±0.007	0.66 ±0.003	0.29 ±0.036
	299	0.99 ±0.017	7.07 ±0.074	0.16 ±0.002	4.37 ±0.055	6.57 ±0.149	13.2 ±0.027	10.85 ±0.114	1.25 ±0.037	1.7 ±0.11	0.65 ±0.003	0.35 ±0.015
	308.13	0.94 ±0.022	8.33 ±0.119	0.17 ±0.001	1.24 ±0.012	4.65 ±0.064	8.46 ±0.097	6.97 ±0.153	1.1 ±0.06	1.84 ±0.09	0.7 ±0.013	0.26 ±0.051

The results are presented as mean value ± standard deviation (n = 3).

**Table 2:** Correlation matrix of the analyzed elements in the inner and outer fraction of contaminated seeds.

	Cr	Mn	Co	Ni	Cu	Zn	Fe	Ca	K	Mg	Na
Cr	**1**	**0.702**	0.564	0.433	0.150	**0.822**	−0.194	−0.410	0.054	−0.191	−0.245
Mn	**0.696**	**1**	**0.749**	0.479	0.293	**0.727**	−0.039	−0.194	0.571	0.263	−0.182
Co	0.400	0.233	**1**	0.517	0.359	**0.797**	0.078	−0.162	0.069	0.278	−0.117
Ni	−0.140	0.228	0.526	**1**	**0.625**	**0.751**	0.396	−0.054	0.213	0.215	0.132
Cu	−0.046	−0.238	−0.083	0.218	**1**	0.328	**0.818**	**0.703**	0.281	**0.776**	**0.790**
Zn	0.469	**0.818**	0.212	0.366	−0.360	**1**	−0.007	−0.403	0.033	−0.012	−0.256
Fe	0.014	−0.161	0.190	0.264	**0.725**	−0.494	**1**	**0.784**	0.237	**0.585**	**0.836**
Ca	−0.123	−0.500	−0.238	−0.338	**0.605**	**−0.777**	0.492	**1**	0.231	**0.790**	**0.949**
K	−0.325	−0.360	0.059	0.292	0.395	−0.516	**0.630**	0.296	**1**	0.440	0.138
Mg	−0.319	−0.547	−0.078	−0.113	**0.641**	**−0.715**	**0.644**	**0.663**	0.253	**1**	**0.694**
Na	−0.060	−0.452	−0.264	−0.488	0.455	**−0.739**	0.347	**0.936**	0.223	**0.607**	**1**

The values of Pearson’s correlation coefficient greater than 0.497 ≈ 0.5 and lower than −0.497 ≈ −0.5 were considered to imply statistically significant correlation (IF—light gray; OF—dark gray) between variables at the predefined significance of *p* = 0.05 (bolded).

## Data Availability

The original contributions presented in the study are included in the article/Supplementary Materials; further inquiries can be directed to the corresponding author.

## Supplementary Materials

The following supporting information can be downloaded at: https://www.mdpi.com/article/10.3390/foods14203552/s1, Table S1. Results of the determination of elements in the reference material ERM-CD281 (rye grass); Table S2. Analytical performance of each element determination; Table S3. Comparison results of mean values for micro- and macroelements between different AFB1 (μg kg^−1^) concentrations in IFs and OFs, respectively. Different letters indicate significant differences according to two-way ANOVA followed by Duncan’s post hoc test. Mean values of the elements between IFs and OFs are significantly different for all AFB1 concentrations.
