# Supplementary material for: Associations Between Mineral Composition and Aflatoxin B1 in Maize (Zea mays L.) Seeds: Toward Contamination Indicators and Food Safety
Source: Foods. 2025 Oct 18;14(20):3552. doi: 10.3390/foods14203552 (PMC12564624; doi:10.3390/foods14203552)
Supplement: Supplementary file 1 [file foods-14-03552-s001.zip › foods-3873699-supplementary.pdf]

Supplementary material

Associations Between Mineral Composition and Aflatoxin B1 in Maize (*Zea mays* L.) Seeds: Toward Contamination Indicators and Food Safety

Dragana Bartolić [1,2,*], Rada Baošić [3], Jelena Mutić [3], Mira Stanković [1,2], Dragosav Mutavdžić [1], Nevena Preradović [1], Saša Krstović [4] and Ksenija Radotić [1,2]

[1] Institute for Multidisciplinary Research (IMSI), University of Belgrade, 11030 Belgrade, Serbia; mira.mutavdzic@imsi.bg.ac.rs (M.S.); gane@imsi.bg.ac.rs (D.M.); nevena@imsi.bg.ac.rs (N.P.); xenia@imsi.bg.ac.rs (K.R.)
[2] Center for Green Technologies, Institute for Multidisciplinary Research (IMSI), University of Belgrade, 11030 Belgrade, Serbia
[3] Faculty of Chemistry, University of Belgrade, Studentski trg 12-16, P.O. Box 51, 11158 Belgrade, Serbia; rbaosic@chem.bg.ac.rs (R.B.); jmutic@chem.bg.ac.rs (J.M.)
[4] Department of Animal Science, Faculty of Agriculture, University of Novi Sad, 21102 Novi Sad, Serbia; sasa.krstovic@stocarstvo.edu.rs
* Correspondence: dragana.bartolic@imsi.bg.ac.rs

Table S1. Results of determination of elements in reference material ERM-CD281 (rye grass)

Element	ERM-CD281 (rye grass)	
	Certified value ± uncertainty* (mg/kg)	Found value ± uncertainty (mg/kg)
Cr	24.8 ± 1.3	25.0 ± 0.2
Cu	10.2± 0.5	10.3 ± 0.3
Mn	82 ± 4	78 ± 7
Ni	15.2 ± 0.6	15.3 ± 0.2
Zn	30.5±1.1	30.0 ± 0.7
	Additional material information (g/kg)	Found value (g/kg)
Fe	0.18	0.181 ± 0.009
Ca	6.3	6.29 ± 0.05
K	34	34.02 ± 0.07
Mg	1.6	1.60 ± 0.09
Na	4.0	4.010 ± 0.003

* Uncertainty for 95 % confidence level (coverage factor k = 2)

Table S2. Analytical performance of each element determination

Element	Correlation Coefficient (r)	Working range (µg/L)	LOD* (µg/L)
Cr	0.999	1-500	0.2
Cu	0.999	10-500	0.9
Mn	0.999	10-500	1.0
Ni	0.999	10-500	1.3
Zn	0.999	10-1000	1.5
	Correlation Coefficient (r)	Working range (mg/L)	LOD* (mg/L)
Fe	0.998	0.1 – 10	0.02
Ca	0.998	0.1 – 10	0.01
K	0.997	0.1 – 10	0.05
Mg	0.997	0.1 – 10	0.01
Na	0.998	0.1 – 10	0.05

**Limit of detection (LOD) was based on 3σ criterion for 10 blank measurements*

Table S3. Results of comparisons of mean values micro and macroelements between different AFB1 (µg kg^{-1}) concentrations separately in IF and OF. Different letters indicate significant differences according to two-way ANOVA followed by Duncan's post hoc test. Mean values of the elements between IF and OF fractions are significantly different for all AFB1 concentrations.

**AFB1**	**Cr**		**Mn**		**Co**		**Ni**		**Cu**		**Zn**		**Fe**		**Ca**		**K**		**Mg**		**Na**	
	IF	OF	IF	OF	IF	OF	IF	OF	IF	OF	IF	OF	IF	OF	IF	OF	IF	OF	IF	OF	IF	OF
**Control**	a	a	c	b	h	c	h	e	h	f	c	b	h	g	d	g	e	abc	g	f	bc	g
**6.75**	b	a	a	a	g	a	e	a	c	a	a	a	e	ab	e	de	cde	a	de	c	cd	abc
**13.26**	a	b	d	cd	a	b	b	h	e	d	b	c	b	bc	f	de	bcd	cd	c	d	e	bcd
**17.07**	c	c	b	d	f	e	a	f	ef	h	d	e	b	f	e	h	bcd	ab	f	i	e	g
**51.51**	d	d	ef	c	c	a	d	b	i	e	e	d	g	d	f	f	de	bc	f	e	de	f
**61.00**	e	d	h	c	d	e	f	d	g	b	f	g	f	c	ef	a	bc	ab	e	b	de	a
**105.00**	c	f	i	e	b	d	f	e	a	c	gh	f	c	a	a	ab	de	ab	b	a	a	abcd
**151.94**	c	f	f	i	ef	e	g	g	d	g	j	j	a	e	a	ab	bcd	e	a	h	a	bcd
**177.00**	c	f	g	g	e	g	f	i	d	g	i	h	a	d	ab	e	a	ab	d	fg	ab	cdf
**248.00**	c	d	e	f	ef	h	d	j	d	e	g	h	a	ab	a	bc	ab	ab	c	e	a	bcd
**299.00**	d	f	j	h	i	f	i	k	f	h	k	i	b	fg	c	de	bcd	cd	b	e	ab	df
**308.13**	f	e	k	j	i	i	c	c	b	d	h	g	d	a	bc	cd	bc	d	b	g	bc	ab
